# Construction of a high-density genetic map by specific locus amplified fragment sequencing (SLAF-seq) and its application to Quantitative Trait Loci (QTL) analysis for boll weight in upland cotton (*Gossypium hirsutum*.)

**DOI:** 10.1186/s12870-016-0741-4

**Published:** 2016-04-11

**Authors:** Zhen Zhang, Haihong Shang, Yuzhen Shi, Long Huang, Junwen Li, Qun Ge, Juwu Gong, Aiying Liu, Tingting Chen, Dan Wang, Yanling Wang, Koffi Kibalou Palanga, Jamshed Muhammad, Weijie Li, Quanwei Lu, Xiaoying Deng, Yunna Tan, Weiwu Song, Juan Cai, Pengtao Li, Harun or Rashid, Wankui Gong, Youlu Yuan

**Affiliations:** State Key Laboratory of Cotton Biology, Key Laboratory of Biological and Genetic Breeding of Cotton, The Ministry of Agriculture, Institute of Cotton Research, Chinese Academy of Agricultural Sciences, Anyang, 455000 Henan China; Biomarker Technologies Corporation, Beijing, 103100 China; Anyang Institute of Technology, Anyang, 455000 Henan China

**Keywords:** Upland cotton (*Gossypium hirsutum* L.), Quantitative trait loci mapping, Specific locus amplified fragment sequencing, Boll weight, Single nucleotide polymorphism marker

## Abstract

**Background:**

Upland Cotton (*Gossypium hirsutum*) is one of the most important worldwide crops it provides natural high-quality fiber for the industrial production and everyday use. Next-generation sequencing is a powerful method to identify single nucleotide polymorphism markers on a large scale for the construction of a high-density genetic map for quantitative trait loci mapping.

**Results:**

In this research, a recombinant inbred lines population developed from two upland cotton cultivars 0–153 and sGK9708 was used to construct a high-density genetic map through the specific locus amplified fragment sequencing method. The high-density genetic map harbored 5521 single nucleotide polymorphism markers which covered a total distance of 3259.37 cM with an average marker interval of 0.78 cM without gaps larger than 10 cM. In total 18 quantitative trait loci of boll weight were identified as stable quantitative trait loci and were detected in at least three out of 11 environments and explained 4.15–16.70 % of the observed phenotypic variation. In total, 344 candidate genes were identified within the confidence intervals of these stable quantitative trait loci based on the cotton genome sequence. These genes were categorized based on their function through gene ontology analysis, Kyoto Encyclopedia of Genes and Genomes analysis and eukaryotic orthologous groups analysis.

**Conclusions:**

This research reported the first high-density genetic map for Upland Cotton (*Gossypium hirsutum*) with a recombinant inbred line population using single nucleotide polymorphism markers developed by specific locus amplified fragment sequencing. We also identified quantitative trait loci of boll weight across 11 environments and identified candidate genes within the quantitative trait loci confidence intervals. The results of this research would provide useful information for the next-step work including fine mapping, gene functional analysis, pyramiding breeding of functional genes as well as marker-assisted selection.

**Electronic supplementary material:**

The online version of this article (doi:10.1186/s12870-016-0741-4) contains supplementary material, which is available to authorized users.

## Background

Upland cotton (*Gossypium hirsutum* L., 2n = 52) is widely grown because it provides superior natural fiber for the textile industry and daily life [1–3]. Increased industrial demand for the fiber makes it a challenge for cotton breeders to increase their yield. Boll weight is one of the important yield components of cotton. But cotton breeders struggle to increase their yield without compromising other fiber traits [4]. Through molecular marker assisted selection (MAS) we can directly select the plants through their genotype. Based on the construction of genetic linkage maps, further studies from identifying the quantitative trait loci (QTLs) of the target traits to identifying the functioning genes, to pyramiding breeding, could be facilitated. Based on MAS, the breeding efficiency could be improved while the breeding cycle is shortened. For the MAS, the density and quality of the genetic map is very important since it forms the basis for the next set of research activities including the detection of reliable and concise QTL confidence intervals, further identification of the functional genes in these concise confidence intervals. Currently most of the genetic maps are based on the simple sequence repeat (SSR) markers with low resolutions. The low polymorphic rate of SSR markers makes it difficult to construct a saturated SSR-based genetic map that covers the whole genome. With the development of the molecular markers, the single nucleotide polymorphism (SNP) markers became widely applied to genetic map construction and MAS due to its large number with a high density across the whole genome. Thus, it is a powerful tool to construct a high-density genetic map (HDGM) and to identify QTLs [5, 6].

The next-generation sequencing (NGS) technique can be used to detect large quantities of SNP markers in the whole genome [7]. There are several methods of NGS including restriction site-associated DNA sequencing (RAD-Seq) [8], Genotyping-by-sequencing (GBS-Seq) [9] and specific locus amplified fragment sequencing (SLAF-seq) [10]. The common feature of these methods is that one or more kinds of restricted DNA-endonuclease(s) were applied to the genome DNA based on the characteristics of the genomes of different species to build a reduced representation library (RRL) of genomic DNA without knowing the detailed information of the whole genome. Thus, each of these methods of NGS was used to construct the HDGM of several species [7, 11, 12]. Zhang et al. [13] constructed an HDGM of *Prunus mume* using SLAF-Seq. The map linked 8007 makers and spanned 1550.62 cM in length with an average marker distance of 0.195 cM. Xu et al. [14] also construct an HDGM of *Cucumis sativus* using SLAF-Seq. The map included 1892 markers with a total distance of 845.7 cM and an average distance of 0.45 cM between adjacent markers. Li et al. [15] construct an HDGM of *Glycine max* with 5785 markers, with a total distance of 2255 cM and an average marker distance of 0.43 cM. Wang et al. [4] constructed an HDGM of cotton using the RAD-Seq method and the map linkage 3984 markers with a total distance of 3499.69 cM.

In this study, a recombinant inbred line (RIL) population, containing 196 individuals was developed from an intra-specific cross between two upland cotton 0–153 and sGK9708. We attempted to use this population to construct an intra-specific HDGM of upland cotton, to identify QTLs and possibly, the candidate genes correlated to cotton boll weight. Finally, a total 5521 SNP markers were successfully applied to genotype these 196 RILs along with parents and an intra-specific HDGM was thus constructed. This map was used to identify QTLs for cotton boll weight across 11 environments.

## Methods

### Plant materials

The intra-specific F_6:8_ recombinant inbred lines (RIL) population of upland cotton with 196 individuals was developed from a cross between homozygous cultivars 0–153 and sGK9708. Cultivar 0–153 harbored superior fiber quality traits while sGK9708 was derived from CRI41 which maintained high yield potential and wide adaptability. The details of the development of RILs have been already described by Sun et al. [16]. Additionally, the phenotypic evaluations of the RILs from 2007 to 2013 were detailed by Zhang et al. [17].

### Phenotypic data analysis

Thirty normally opened bolls within five to eight fruiting branches and one to three fruiting nodes were sampled in annually September. The total seed-cotton of the 30 bolls was weighted and average boll weight was calculated accordingly. One-way ANOVA was used to test the significance of the differences in boll weight between two parents. Additionally, EXCEL 2010 was used to create the descriptive statistics including the mean value, standard deviation, skewness and kurtosis of the boll weight across the whole population.

### DNA extractions and SLAF library construction and high-throughput sequencing

The leaves of the parents and the RIL population were sampled in July and stored at −70 °C. The genomic DNA was extracted using the TaKaRa MiniBEST Plant Genomic DNA Extraction kit (TaKaRa, Dalian) and SLAF-seq strategy with some modifications was utilized in the library construction. Briefly, the reference genome of *Gossypium hirsutum* [18, 19] was referred to make the pre-experiment *in silico* simulation of the number of markers generated by various endonuclease combinations. The SLAF library was constructed based on the SLAF pilot experiment in accordance with the predesigned scheme and eventually two endonucleases combination of *Hae*III and *Ssp*I (New England Biolabs, NEB, USA) was applied to the genomic DNA digestion in our RIL population. The details of SLAF-seq strategy was described by Zhang et al. [13].

### Grouping and genotyping of sequencing data

SLAF markers were identified and genotyped with procedures described by Sun et al. [10] and Zhang et al. [13]. Briefly, after filtering out the low-quality reads (quality score < 20e), the remaining reads were sorted to each progeny according to duplex barcode sequences. Then each of the high-quality read was trimmed off 5-bp terminal position. Finally 80 bp pair-end clean reads were obtained from the same sample and were mapped onto the genome of *Gossypium hirsutum* [19] sequence using BWA software [20]. Sequences mapping to the same position with over 95 % identity were defined as one SLAF locus [13]. SNP loci in each SLAF locus were then detected between parents using the software GATK. SLAFs with more than three SNPs were filtered out first. As the sequenced size of the fragments was only 160 bp, three or more SNPs in one SLAF indicated a significantly high heterozygosity of upland cotton (more than 1 %). This would lead to a decreased accuracy and reliability of the sequencing and genotyping. The SLAFs were genotyped depending on the tags of the parents sequenced above tenfold depth and the individuals of the RIL population were genotyped based on the similarity to the parents. As each SLAF locus harbored at most three SNP loci, it was possible that one SLAF locus could harbor at most, four SLAF alleles. The SLAF repetitiveness and polymorphism were defined based on the criteria described by Zhang et al. [13]. The repetitive SLAFs were discarded and only the polymorphic SLAFs were considered as potential markers. Only the SLAFs with consistency in the parental and RIL were genotyped.

The procedure of all polymorphic SLAF loci genotyping was described by Sun et al. [10] and Zhang et al. [13]. Before genetic map construction, all the SLAF markers were filtered using a criteria detailed by Zhang et al. [13] besides the markers with more than 40 % missing data were filtered out.

### Linkage map construction

Linkage map was constructed based on the procedure detailed by Zhang et al. [13] and the cotton genome database [19]. HighMap strategy for ordering the SLAF and correcting genotyping errors within the chromosomes was detailed by Liu et al., Jansen et al. and van Ooijen et al. [21–23]. SMOOTH was also applied to the error correction strategy according to parental contribution to the genotypes of the progeny [24], and a k-nearest neighbor algorithm was used to impute the missing genotypes [25]. A multipoint method of maximum likelihood was applied to add the skewed markers into the linkage map. The Kosambi mapping function was applied to estimate the map distances [26].

### Segregation distortion analysis

As the distortedly segregated markers showing significance between 0.001 and 0.05 (0.001 < *p* < 0.05) were still maintained to construct the HDGM, the region on the map with more than three consecutive adjacent loci that showed significant (0.001 < *P* < 0.05) segregation distortion was defined as a segregation distortion region (SDR) [11]. The size and distribution of SDRs on the map were analyzed.

### Collinearity and recombination hotspot analysis

All the sequences of SNP markers that were constructed in the linkage map were aligned back to the physical sequence of the upland cotton genome through local Basic Local Alignment Search Tool (BLAST) to confirm their physical positions in the genome. Software CIRCOS 0.66 was used to compare the collinearity of markers based on their genetic positions and physical positions. The recombination hotspot (RH) was estimated based on the recombination rate of markers. If the value that the genetic distance between adjacent markers was divided by was higher than 20 cM/Megabase, the region between the two adjacent markers was regarded as RH [13].

### QTL analysis using HDGM

Windows QTL Cartographer 2.5 [27] was used to identify QTLs by composite interval mapping method [28] on the environment by environment basis of the 11 environments. The LOD threshold for declaring significant QTLs included the QTLs across environments calculated by a permutation test with the mapping step of 1.0 cM, five control markers, and a significance level of *P* < 0.05, *n* = 1000. LOD score values between 2.0 and permutation test LOD threshold were used to declare suggestive QTL. Positive additive effect means that the favorable alleles come from the 0–153 parent while negative additive effect means that the favorable alleles come from sGk9708. QTLs were named and the common QTLs were identified as described by Sun et al. [16].

### The candidate genes identification

The markers flanking the confidence intervals of the QTLs which can be detected in at least three environments were selected to identify the candidate genes. The sequences of these markers were aligned back to the physical sequence of upland cotton genome database [19]. Based on the position of these flanking markers, all the genes within the confidence interval were identified as candidate genes. For some of the QTLs with a large confidence interval, if the position of one marker flanking the confidence interval was too far from that of the nearest marker harbored in that confidence interval, the region between these two markers was excluded from the candidate gene identification. All the candidate genes were categorized through the gene ontology (GO) analysis. The first ten terms that have the smallest Kolmogorov-Smirnov (KS) values were considered as the enriched terms. The pathways correlated to the candidate genes were discovered by the Kyoto Encyclopedia of Genes and Genomes (KEGG) analysis. The first ten pathways with the smallest *p* values were considered as the enriched pathways. The candidate genes were also categorized based on their products through eukaryotic orthologous groups (KOG) database analysis.

## Result

### Performance of boll weight of RIL populations

The one-way ANOVA result showed the *p*-value was 0.002, suggesting that significant differences of boll weight were found between the two parents. The descriptive statistical analysis results of the RIL population and parents across 11 environments were shown in Table 1. The absolute value of skewness of the mean value of the boll weight in the RIL population across 11 environments was less than one, indicating an approximately normal distribution. In all 11 environments, both the positive transgressive segregation (the observed values are higher than that of sGK9708) and the negative transgressive segregation (the observed values are lower than that of 0–153) of the boll weight in the RIL population were observed (Table 1).

**Table 1:** The results of the statistical analysis of the parents and the whole population

Env	Parents				Population							
	0–153	SGK9708	Range	*P*-value	Min	Max	Range	Average	Std.Sdv	Var	Skew	Kurt
07ay	4.46	5.18	0.71	0.0021	3.92	5.91	1.99	4.71	0.41	0.17	0.38	0.05
08ay	4.49	5.74	1.24		3.50	6.20	2.70	4.78	0.47	0.22	0.06	0.42
08lq	4.40	5.72	1.32		3.97	6.29	2.32	4.91	0.47	0.22	0.35	−0.16
08qz	3.85	4.77	0.92		3.20	5.50	2.30	4.32	0.47	0.22	0.03	−0.56
09ay	3.56	4.65	1.09		2.99	5.40	2.41	4.15	0.44	0.19	0.14	−0.02
09qz	2.93	4.44	1.51		2.13	5.16	3.03	3.41	0.55	0.30	0.14	−0.39
09xj	5.20	5.40	0.20		3.73	6.94	3.21	5.17	0.57	0.32	0.15	0.24
10gy	3.20	3.79	0.59		1.78	4.65	2.87	3.40	0.48	0.23	−0.16	−0.04
10ay	4.20	5.44	1.24		3.32	5.83	2.51	4.61	0.48	0.23	0.09	−0.17
10zz	3.71	5.98	2.27		2.38	5.86	3.48	3.94	0.57	0.33	0.06	0.45
13ay	5.13	5.62	0.49		2.76	6.26	3.50	4.70	0.55	0.30	−0.24	0.86

### Analysis of SLAF-seq data and SLAF markers

**Fig. 1:**
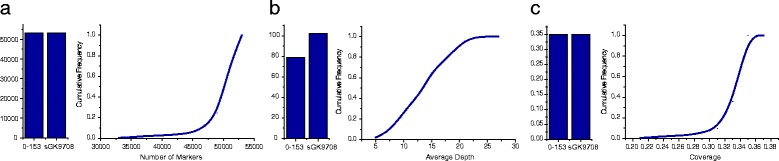
The information of sequencing data in each line in the whole RIL population. **a** Distribution of the number of markers in each line of the whole RIL population. **b** Distribution of the average sequencing depths in each line of the whole RIL population. **c** Distribution of the coverage in each line of the whole RIL population

After SLAF library construction and sequencing, 87.89 GB of data containing 443.56 M pair-end reads was generated with each read of 80 bp in length. Among them, 82.24 % of the bases were of high quality with Q20 (means a quality score of 20, indicating a 1 % chance of an error, and thus 99 % confidence) and guanine-cytosine (GC) content was 34.47 %. The SLAFs numbers of 0–153 and sGK9708 were 53,123 and 53,238, and their correspondent sequencing depths were 78.66 and 102.13 respectively. The coverage of both parents was 35 %. In the RIL population, the number of SLAFs ranged from 32,261 to 53,104 and the average number of SLAFs was 50,487. The average sequencing depth was 14.50, and the average coverage was 33.37 % (Fig. 1).

The 443.56 M pair-end reads, consisting of 53,754 SLAFs, totally harbored 160,876 SNP markers, as usually one SLAF can harbor more than one and at most three SNP markers. Among the 160,876 SNP markers, 23,519 markers were identified polymorphic across the whole RIL population with a polymorphic rate of 14.62 %. All the polymorphic SNP markers were classified into four genotypes: aa × bb, hk × hk, lm × ll and nn × np. The aa × bb meant that both of the parents were homozygous in this SNP position, the genotype of one parent was aa and the other was bb; the hk × hk meant that both of the parents were heterozygosis, and the lm × ll and nn × np meant that one of the parent was heterozygosis and the other was homozygous. Only the genotype aa × bb, consisting of 18,318 SNPs, was used for further analysis. Among 18,318 markers, the marker with average sequence depths less than four were filtered with 16,490 markers left. Then the markers with polymorphism across the whole population but not between parents were excluded leaving 15,076 markers remaining. The 15,076 markers were further filtered by a criterion of more than 40 % missing data and 10,588 markers left. Finally, Markers with significant segregation distortion (*P* < 0.001) were filtered and the remaining 5521 markers, including the ones that showed significant segregation distortion between 0.05 and 0.001 (0.001 < *P* < 0.05) were used to construct the final genetic map (Table 2).

**Table 2:** The whole process of filtering markers

Filtered step	Number
All the Reads	443.65 MB
The Reads of High Quality with Q20	364.86 MB
SLAFs in the Reads	53,754
SNPs in the SLAFs	160,876
Polymorphic SNPs across the Whole RIL Population	23,519
SNPs of AA × BB Genotype	18,318
Deep of SNPs More Than Four	16,490
Polymorphic SNPs between parents	15,076
Percentage of Missing Data less than 40 %	10,588
SNPs with non segregation distortion (*p* ≥ 0.05) and with significant segregation distortion (0.001 < *P* < 0.05)	5521

### Distribution of SNP markers’ type on the genetic map

In total, 5521 SNP loci were mapped on the final linkage map and percentages of SNP types were investigated (Additional file 1: Table S1). Most of the SNPs were transitions of Thymine (T)/Cytosine (C) and Adenine (A)/Guanine (G), accounting for 34.49 and 33.74 % of all SNP markers respectively. The other four SNP types were transversions including G/C, A/C, G/T and A/T with percentages of 4.46, 8.08, 8.35 and 10.89 % respectively and collectively accounted for 31.77 % of all SNPs (Additional file 1: Table S1).

### Construction of the genetic map

**Fig. 2:**
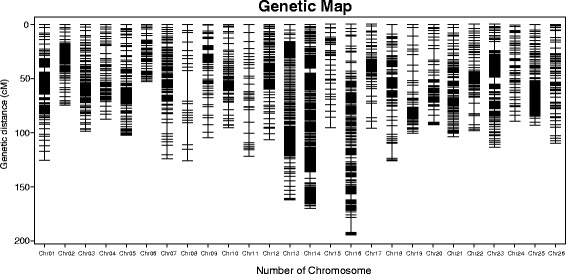
The genetic map constructed by SNP markers

The map harbored 5521 SNP markers, spanning a total distance of 3259.37 cM with an average marker interval of 0.78 cM. The A sub-genome harbored 3550 markers with a total distance of 1838.37 cM whereas the D sub-genome harbored 1971 markers with a total distance of 1421 cM. The largest chromosome was chromosome 05, which contained 434 markers with a genetic length of 242.56 cM, and an average marker interval of 0.56 cM. The shortest chromosome was chromosome 15, which only harbored 29 markers with a genetic length of 41.39 cM and an average marker interval of 1.43 cM. The largest gap on this map was only 7.02 cM located on chromosome 26. There were totally 11 gaps greater than 5.00 cM, three of which were on chromosome 10 and with remaining eight on eight different chromosomes. The remaining chromosomes had no visible gaps (Additional file 2: Table S2, Fig. 2, Table 3).

**Table 3:** The detail information of the high-density genetic map

Chromosome number	Marker number	Total distance	Average distance	Largest gap	Number of gap (>5 cM)	Number of SDMs	Percentage of SDMs	X^2^_value	*P*_value	SDR region	Number of RHs
Chr01	297	140.42	0.47	4.48	0	82	27.61 %	2.50	0.28	9	32
Chr02	180	136.88	0.76	5.42	1	12	6.67 %	1.36	0.44	1	35
Chr03	218	159.93	0.73	4.15	0	47	21.56 %	2.36	0.40	4	0
Chr04	574	142.01	0.25	3.61	0	27	4.70 %	1.14	0.43	0	86
Chr05	434	242.56	0.56	4.22	0	106	24.42 %	2.46	0.29	10	0
Chr06	101	92.62	0.92	4.76	0	26	25.74 %	2.37	0.43	1	16
Chr07	318	132.96	0.42	3.56	0	36	11.32 %	1.58	0.35	1	1
Chr08	56	45.12	0.81	3.56	0	13	23.21 %	2.32	0.26	0	0
Chr09	274	156.33	0.57	5.07	1	60	21.90 %	2.30	0.32	5	55
Chr10	133	113.33	0.85	6.69	3	17	12.78 %	1.86	0.32	1	32
Chr11	88	112.62	1.28	5.71	1	24	27.27 %	2.50	0.30	3	0
Chr12	273	178.26	0.65	5.07	1	85	31.14 %	2.85	0.28	8	37
Chr13	604	185.33	0.31	4.15	0	44	7.28 %	1.43	0.40	1	106
Chr14	408	173.03	0.42	4.46	0	238	58.33 %	4.98	0.18	18	67
Chr15	29	41.39	1.43	3.56	0	8	27.59 %	2.76	0.33	1	1
Chr16	399	178.54	0.45	3.61	0	152	38.10 %	3.38	0.28	13	0
Chr17	102	101.64	1	4.79	0	9	8.82 %	1.28	0.43	0	29
Chr18	172	136.45	0.79	5.07	1	43	25.00 %	2.67	0.27	3	1
Chr19	109	94.13	0.86	4.76	0	18	16.51 %	2.10	0.35	2	24
Chr20	60	48.44	0.81	4.15	0	9	15.00 %	2.27	0.28	0	11
Chr21	174	163.73	0.94	5.71	1	29	16.67 %	1.77	0.43	2	40
Chr22	75	65.91	0.88	4.46	0	4	5.33 %	1.22	0.50	0	14
Chr23	142	127.61	0.9	4.76	0	31	21.83 %	2.26	0.32	3	36
Chr24	60	76.99	1.28	4.76	0	6	10.00 %	1.39	0.47	0	12
Chr25	166	124.21	0.75	5.39	1	84	50.60 %	4.62	0.16	6	39
Chr26	75	88.93	1.19	7.02	1	15	20.00 %	2.13	0.35	1	19
Total	5521	3259.37	0.78	7.02	11	1225	--	--	--	93	693

### The quality analysis of the high-density genetic map

In total, 1225 markers of the mapped 5521 showed significant (0.05 < *P* < 0.001) segregation distortion. These segregation distortion markers (SDMs) were located in the chromosomes with an uneven distribution in each. Among the 1225 SDMs, 579 of them were located in the A subgenome of upland cotton whereas 646 of them were located in the D subgenome of upland cotton. Chromosome 14 had the largest number of SDMs and accounted for the highest percentage of SDMs of all the mapped markers. The number of SDMs on c14 was 238 and accounted for 58.33 % of the total markers mapped on it. Chromosome 22 had the smallest number of SDMs (four). Chromosome 4 had 4.7 % SDMs, the lowest overall percentage. In total, 93 SDRs were defined in all the chromosomes, with 44 of them located in the A subgenome of upland cotton and the other 49 located in the D subgenome of upland cotton. Chromosome 14 had the most SDR number, 18 SDRs, while chromosomes 4, 8, 17, 20, 22, and 24 had no SDR (Additional file 3: Table S3, Table 3).

**Fig. 3:**
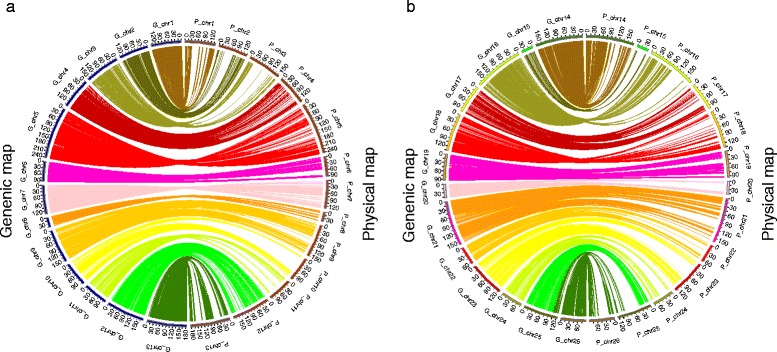
Collinearity between the genetic map and the physical map. **a** Collinearity of the A sub-genome between the genetic map and the physical map. **b** Collinearity of the D sub-genome between the genetic map and the physical map

Collinearity analysis of the SNP loci between the genetic map and the physical map is shown in Fig 2. The results indicated that the genetic map constructed by the SNP markers which were discovered through SLAF-seq had a sufficient coverage over the cotton genome. Most of the SNP loci on the linkage map were in same order as those on the corresponding chromosomes of the physical map of the cotton genome. D subgenome showed a better compatibility with the physical map as compared to the A subgenome. Chromosomes 1, 2, 3, 5, 7, and11 in the A subgenome and chromosomes 14, 15, 16 and 18 in the D subgenome showed some deviation in collinearity analysis (Additional file 4: Table S4, Fig. 3).

**Fig. 4:**
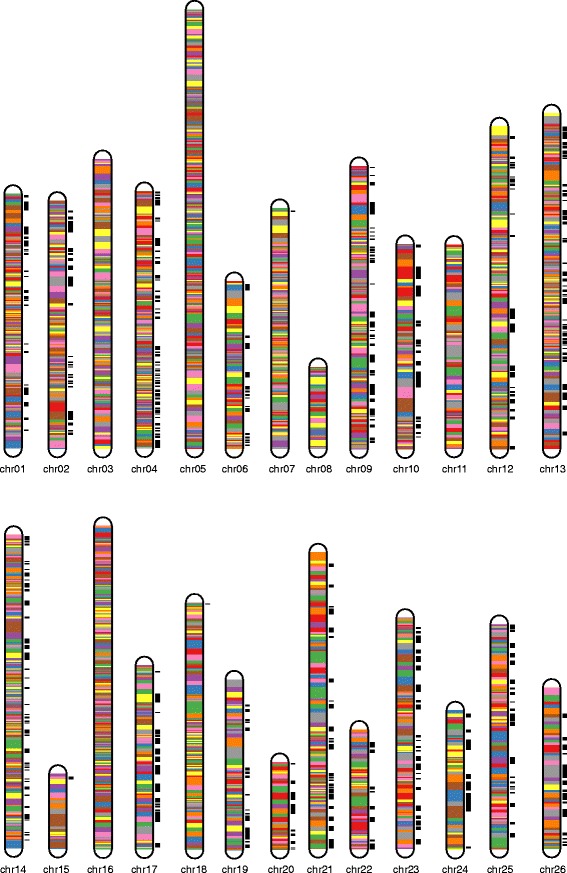
The genetic position of the recombination hotspots in the whole 26 chromosomes

The result of the RH analysis showed that among the 26 chromosomes, 21 have RHs, 9 and 12 of which were in the A subgenome and D subgenome respectively. Chromosome 13 harbored the largest number of 106 RHs whereas the chromosomes 7, 15 and 18 only harbored one RH. Chromosomes 3, 5, 8, 11 and 16 did not harbor any RH. Additional information is shown in Additional file 5: Table S5, Fig. 4, and Table 3.

### QTL mapping for boll weight in the RILs

**Fig. 5:**
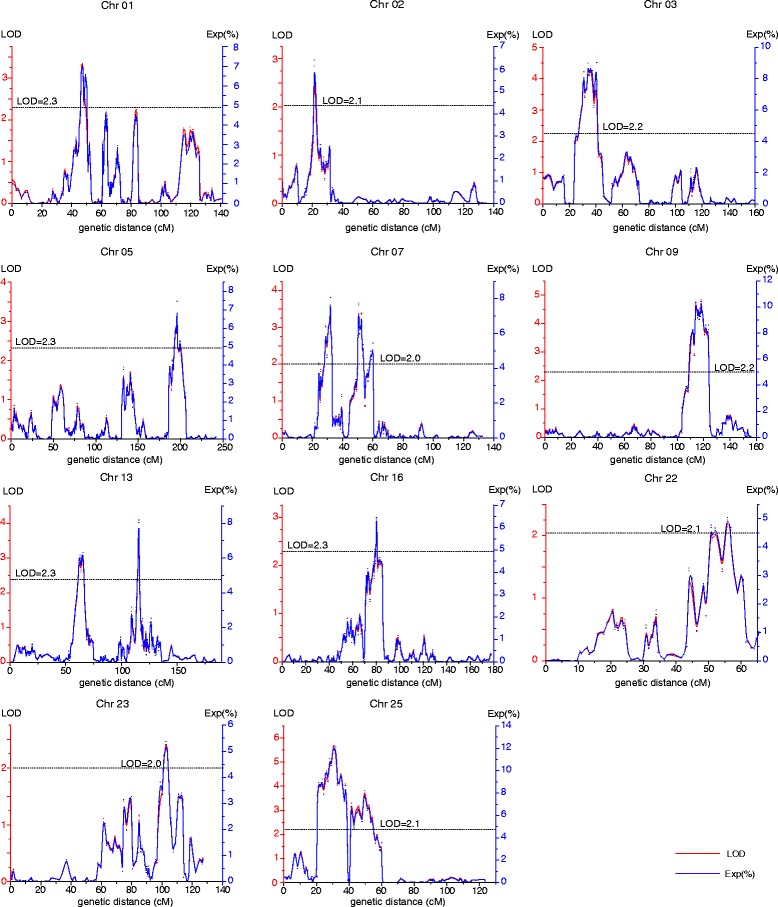
The LOD value and the observed PV value of the stable QTLs

A total of 146 QTLs for boll weight trait were detected on 25 chromosomes across 11 environments (chromosome 8 was the exception). Sixteen of them were regarded as stable QTLs as they could be detected in at least three environments. In the confidence intervals of these stable QTLs, *qBW-chr13-7* harbored 26 markers whereas *qBW-chr02-3* and *qBW-chr25-6* only harbored two markers. Among these stable QTLs, *qBW-chr13-7*, detected in seven environments, was located within the marker interval of CRI-SNP8685-CRI-SNP8731, and could explain 6.13–14.70 % of the observed phenotypic variation (PV). QTL *qBW-chr13-4*, detected in six environments, was located within the marker interval of CRI-SNP8313-CRI-SNP-8346, and explained 4.58–6.06 % of the observed PV. QTLs *qBW-chr01-1* and *qBW-chr25-5*, both of which were detected in five environments, were located within the marker intervals of CRI-SNP147-CRI-SNP168 and CRI-SNP10564-CRI-SNP10569, and explained 4.81–7.83 % and 4.29–10.76 % of the observed PV respectively. QTLs *qBW-chr02-3, qBW-chr07-1, qBW-chr07-6, qBW-chr09-6* and *qBW-chr25-7*, all of which were detected in four environments, located within the marker intervals of CRI-SNP506-CRI-SNP519, CRI-SNP-5634-CRI-SNP5581, CRI-SNP5454-CRI-SNP-5438, CRI-SNP6432-CRI-SNP6455 and CRI-SNP10592-CRI-SNP10615, and explained 5.62–6.41, 4.95–8.89, 5.35–10.89, 5.01–10.31 and 7.58–7.80 % of the observed PV respectively. QTLs *qBW-chr03-1, qBW-chr05-10, qBW-chr07-4, qBW-chr16-4, qBW-chr22-3, qBW-chr23-5* and *qBW-chr25-6*, all of which were detected in three environments, were located within the marker intervals of CRI-SNP-1241-CRI-SNP-1231, CRI-SNP-2294-CRI-SNP-2279, CRI-SNP-5497-CRI-SNP5472, CRI-SNP12560-CRI-SNP12270, CRI-SNP10330-CRI-SNP10341, CRI-SNP13838-CRI-SNP13865 and CRI-SNP10569-CRI-SNP10571, and explained 4.56–9.00, 5.64–7.45, 6.92–8.45, 4.15–5.03, 6.64–8.80, 4.26–5.26 and 4.82–11.85 % of the observed PV respectively (Additional file 6: Table S6, Fig. 5, Table 4, Table 5).

**Table 4:** The detail information about the stable QTLs

QTL name	Environment	Position	LOD	Additive	R2	Marker interval (*P* < 0.01)	Marker interval (*P* < 0.05)	LOD_L (*P* < 0.01)	LOD_R (*P* < 0.01)	LOD_L (*P* < 0.05)	LOD_R (*P* < 0.05)
qBW-chr01-1	10GY	45.41	2.43	0.25	5.32 %	CRI-SNP161-CRI-SNP168	CRI-SNP147-CRI-SNP168	45.10	47.00	44.30	47.70
	07AY	46.41	2.20	0.20	4.81 %			46.00	47.70	45.40	50.30
	08AY	47.41	2.52	0.18	5.19 %			45.10	48.20	42.50	50.30
	08LQ	47.41	3.35	0.19	7.05 %			46.00	49.50	44.70	50.30
	08QZ	47.41	3.44	0.28	7.83 %			45.60	49.20	45.40	50.30
qBW-chr02-3	08AY	21.11	2.82	0.15	6.15 %	CRI-SNP511-CRI-SNP512	CRI-SNP506-CRI-SNP519	20.70	23.00	20.70	25.10
	08LQ	21.11	2.52	0.15	5.62 %			19.70	23.00	18.40	25.10
	08QZ	21.11	2.85	0.16	6.41 %			20.70	22.50	19.40	24.30
	10AY	21.11	2.57	0.15	5.67 %			19.30	23.80	18.40	27.30
qBW-chr03-1	08AY	34.01	4.50	0.16	9.00 %	CRI-SNP-1241-CRI-SNP-1235	CRI-SNP-1241-CRI-SNP-1231	32.60	34.80	31.40	34.80
	08LQ	34.01	3.85	0.16	8.29 %			33.40	35.80	33.20	38.10
	10AY	34.01	2.28	0.11	4.56 %			31.40	36.80	31.40	45.30
qBW-chr05-10	09AKS	195.81	3.52	−0.16	7.45 %	CRI-SNP-2294-CRI-SNP-2279	CRI-SNP-2294-CRI-SNP-2279	195.00	197.50	195.00	197.90
	07AY	199.21	3.50	−0.11	7.43 %			199.00	200.50	197.60	200.50
	13AY	199.21	2.85	−0.16	5.64 %			199.10	200.30	197.60	200.50
qBW-chr07-1	09AKS	31.51	3.97	0.17	8.89 %	CRI-SNP-5633-CRI-SNP5596	CRI-SNP-5634-CRI-SNP5581	30.40	32.10	30.40	32.20
	08AY	32.01	2.85	0.20	6.32 %			31.40	32.80	30.00	33.50
	08QZ	32.01	2.41	0.20	4.95 %			31.40	32.50	31.40	32.50
	09AY	32.01	3.80	0.19	8.07 %			31.40	32.30	29.60	33.00
qBW-chr07-4	13AY	50.61	3.66	−0.24	7.64 %	CRI-SNP5490-CRI-SNP5481	CRI-SNP-5497-CRI-SNP5472	50.10	51.10	49.80	51.10
	09QZ	51.11	3.34	−0.23	6.92 %			50.10	52.30	49.30	53.20
	10AY	51.11	4.08	−0.23	8.45 %			50.30	51.50	50.10	51.60
qBW-chr07-6	10AY	58.61	4.38	−0.22	9.03 %	CRI-SNP5452-CRI-SNP-5441	CRI-SNP5454-CRI-SNP-5438	57.80	59.30	56.80	60.10
	10ZZ	58.61	5.21	−0.28	10.89 %			57.80	59.20	57.80	59.70
	09QZ	59.11	2.55	−0.19	5.35 %			57.80	60.20	57.80	60.70
	13AY	60.21	2.58	−0.19	5.45 %			59.90	60.50	59.90	60.80
qBW-chr09-6	07AY	114.11	4.77	−0.14	10.31 %	CRI-SNP6432-CRI-SNP6455	CRI-SNP6432-CRI-SNP6455	113.70	115.40	112.80	115.40
	09QZ	114.11	2.44	−0.13	5.01 %			113.00	116.70	112.80	116.70
	09AKS	114.11	2.75	−0.16	5.80 %			112.70	114.60	112.00	114.60
	09AY	114.61	3.27	−0.14	6.54 %			112.90	115.40	112.80	115.40
qBW-chr13-4	08LQ	58.71	2.43	−0.12	4.58 %	CRI-SNP8317-CRI-SNP-8338	CRI-SNP8313-CRI-SNP-8346	57.40	60.00	56.10	60.00
	13AY	60.01	2.55	−0.17	5.24 %			58.60	63.10	58.20	66.30
	09AY	62.81	2.33	−0.11	5.05 %			58.10	66.30	57.90	70.10
	07AY	64.51	2.99	−0.12	6.06 %			63.70	66.80	63.70	68.30
	08AY	64.51	2.76	−0.13	5.17 %			63.70	67.80	63.70	68.90
	10AY	64.51	2.46	−0.12	4.87 %			62.10	66.80	58.70	68.90
qBW-chr13-7	09AKS	114.61	2.95	0.34	6.13 %	CRI-SNP8690-CRI-SNP8726	CRI-SNP8685-CRI-SNP8731	113.90	115.90	113.20	116.50
	08LQ	114.91	8.37	0.52	16.70 %			114.60	115.30	114.50	115.50
	08QZ	115.11	7.21	0.50	14.76 %			114.70	116.20	114.50	115.80
	10AY	115.11	4.14	0.38	8.36 %			114.90	115.40	114.60	115.50
	08AY	115.41	6.97	0.49	13.72 %			114.90	115.90	114.90	115.70
	09QZ	115.41	2.99	0.34	6.45 %			114.60	116.30	114.30	117.30
	07AY	115.61	4.03	0.33	8.21 %			115.40	117.10	115.40	116.50
qBW-chr16-4	09AY	80.21	2.97	−0.14	6.46 %	CRI-SNP12560-CRI-SNP12271	CRI-SNP12560-CRI-SNP12270	79.40	81.00	79.40	81.20
	10AY	80.21	4.12	−0.22	8.48 %			79.80	84.30	79.40	83.30
	07AY	83.01	3.25	−0.13	6.85 %			82.00	86.00	82.00	87.00
qBW-chr22-3	09AY	52.61	2.10	−0.10	4.52 %	CRI-SNP10333-CRI-SNP10341	CRI-SNP10330-CRI-SNP10341	51.00	54.20	49.20	56.80
	10GY	55.81	1.97	−0.10	4.15 %			51.00	59.90	55.80	55.80
	10AY	55.81	2.25	−0.11	5.03 %			54.20	58.30	54.20	58.90
qBW-chr23-5	08AY	101.81	2.14	0.12	4.26 %	CRI-SNP13840-CRI-SNP13862	CRI-SNP13838-CRI-SNP13865	98.00	106.50	96.80	107.30
	10ZZ	102.61	2.46	0.16	5.26 %			99.00	105.00	96.90	105.80
	08QZ	103.61	2.40	0.13	5.17 %			100.90	104.70	97.00	105.80
qBW-chr25-5	08AY	22.41	4.39	0.19	9.36 %	CRI-SNP10565-CRI-SNP10569	CRI-SNP10564-CRI-SNP10569	20.40	23.50	20.40	24.40
	10ZZ	22.41	5.17	0.25	10.76 %			20.40	24.20	20.40	26.30
	08LQ	22.51	2.20	0.13	4.29 %			20.40	26.40	20.40	27.10
	09AY	23.51	4.08	0.18	9.26 %			20.40	24.40	20.30	24.40
	09QZ	25.41	2.52	0.17	6.11 %			23.80	29.20	23.50	29.20
qBW-chr25-6	10ZZ	28.11	3.06	0.20	7.08 %	CRI-SNP10569-CRI-SNP10568	CRI-SNP10569-CRI-SNP10571	27.10	32.80	27.10	32.80
	09AY	30.81	5.68	0.21	11.85 %			27.70	32.50	24.40	32.90
	09QZ	30.81	2.17	0.15	4.82 %			29.20	32.50	29.20	32.90
qBW-chr25-7	10GY	45.91	3.51	−0.22	7.79 %	CRI-SNP10592-CRI-SNP10614	CRI-SNP10592-CRI-SNP10615	44.90	47.60	44.40	47.00
	10AY	45.91	3.83	−0.15	7.70 %			44.70	48.00	44.40	48.00
	09AY	49.61	3.83	−0.15	7.80 %			48.30	53.00	48.00	53.50
	10ZZ	52.71	3.63	−0.18	7.58 %			52.50	53.20	52.50	53.20

**Table 5:** The markers and the candidate genes in the confidence intervals of the stable QTLs

QTL name	Marker interval (*P* < 0.01)	Gene interval	Physical distance interval	Number of markers	Number of genes
qBW-chr01-1	CRI-SNP161-CRI-SNP168	CRI-SNP161-CRI-SNP166	21363529–22191102	5	8
qBW-chr02-3	CRI-SNP511-CRI-SNP512	CRI-SNP511-CRI-SNP512	2428231–2465227	2	None
qBW-chr03-1	CRI-SNP-1241-CRI-SNP-1235	CRI-SNP-1241-CRI-SNP-1235	93109282–93363954	6	3
qBW-chr05-10	CRI-SNP-2294-CRI-SNP-2279	CRI-SNP-2294-CRI-SNP-2281	11840100–12807341	11	51
qBW-chr07-1	CRI-SNP-5633-CRI-SNP5596	CRI-SNP-5633-CRI-SNP5596	41686619–43069600	18	15
qBW-chr07-4	CRI-SNP5490-CRI-SNP5481	CRI-SNP5490-CRI-SNP5481	26629060–26694814	10	1
qBW-chr07-6	CRI-SNP5452-CRI-SNP-5441	CRI-SNP5452-CRI-SNP-5441	26153119–26450470	7	11
qBW-chr09-6	CRI-SNP6432-CRI-SNP6455	CRI-SNP6432-CRI-SNP6455	55762226–57316457	15	28
qBW-chr13-4	CRI-SNP8317-CRI-SNP-8338	CRI-SNP8317-CRI-SNP-8338	5157441–5989840	13	34
qBW-chr13-7	CRI-SNP8690-CRI-SNP8726	CRI-SNP8690-CRI-SNP8726	41941944–43033838	26	10
qBW-chr16-4	CRI-SNP12271-CRI-SNP12560	CRI-SNP12483-CRI-SNP12560	15223879–15984482	19	37
qBW-chr22-3	CRI-SNP10333-CRI-SNP10341	CRI-SNP10333-CRI-SNP10341	47103662–47711028	8	39
qBW-chr23-5	CRI-SNP13840-CRI-SNP13862	CRI-SNP13840-CRI-SNP13862	43266988–43944781	7	65
qBW-chr25-5	CRI-SNP10565-CRI-SNP10569	CRI-SNP10565-CRI-SNP10569	1826714–2154361	5	32
qBW-chr25-6	CRI-SNP10569-CRI-SNP10568	CRI-SNP10569-CRI-SNP10568	2129899–2154631	2	1
qBW-chr25-7	CRI-SNP10592-CRI-SNP10614	CRI-SNP10592-CRI-SNP10614	2861896–3087983	10	10

### The candidate genes annotation

**Fig. 6:**
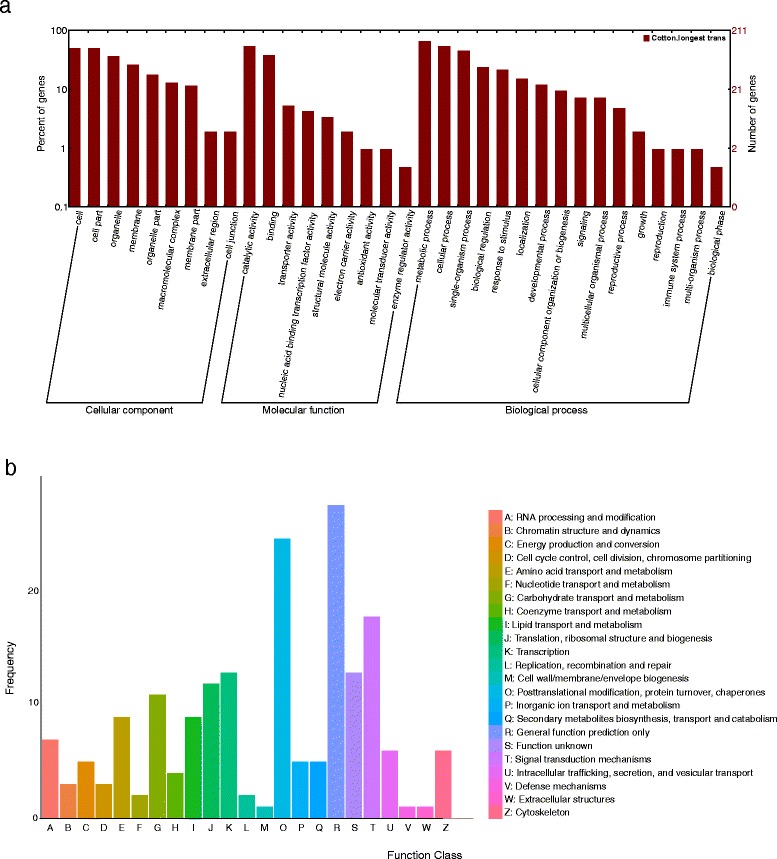
The annotation of the candidate genes in the confidence intervals of the stable QTLs. **a** The annotation of the candidate genes in the confidence intervals of the QTLs that could be detected in at least three environments through GO analysis. **b** The annotation of the candidate genes in the confidence intervals of the QTLs that could be detected in at least three environments through KOG analysis

In total, 344 candidate genes were identified in the confidence intervals of stable QTLs. Except for the confidence interval of *qBW-chr02-3* which has no candidate gene, the confidence intervals of all the remaining QTLs have candidate genes. The confidence intervals of *qBW-chr07-4* and *qBW-chr25-6* harbored only one candidate gene whereas the confidence interval of *qBW-chr23-5* harbored 65 genes (Additional file 7: Figure S1, Additional file 8: Figure S2). In total, 340 of the 344 candidate genes had annotation information, among which 201, 81 and 163 had annotation information in GO, KEGG and KOG respectively. In GO analysis, 435 genes were identified in the cellular component category, 221 genes in the molecular function category, and 549 genes in the biological process category, as some of the genes had multiple functions and could be categorized into two or more function baskets. In the cellular component category, 102 genes were related to cell and 101 genes were related to cell part. In the molecular function category, 108 genes were related to catalytic activity. In the biological process category, 133 genes were related to metabolic process and 108 genes were related to cellular process (Additional file 9: Table S7, Fig. 6). In the KEGG analysis, 81 genes were identified in 55 pathways. Six genes were found in the plant hormone signal transduction pathway, four genes were found in both the ribosome and protein processing pathways in endoplasmic reticulum In all the remaining pathways, there were no more than three genes found (Additional file 10: Table S8, Additional file 11: Table S9). In the KOG analysis, 24 genes only had the general prediction function and 12 genes had unknown function. Among the other 127 genes, 25 of them were related to posttranslational modification, protein turnover, and chaperones, 17 of them had a relation to signal transduction mechanisms, 12 of them had a relation to translation, ribosomal structure and biogenesis, 11 of them had a relation to carbohydrate transport and metabolism and 11 of them had a relation to transcription. No more than 10 genes were found in other functions in KOG classification (Fig. 6, Additional file 12: Table S10, Additional file 13: Table S11, Table 5).

Among all 344 candidate genes, 44 were identified at the nearest positions of the markers, of which the genetic position had the highest LOD values in the QTL mapping analysis (Additional file 7: Figure S1, Additional file 8: Figure S2). Among them, 43 candidate genes had annotation information except the gene *Gh_D06G0216*. In the KEGG analysis, eight cand genes had annotation information, five of which were related to hypothetical protein, with the other three related s-adenosylmethionine synthetase, polygalacturonase precursor and indole-3-acetic acid-amido synthetase GH3.3 respectively. In KOG analysis, 18 candidate genes had annotation information. Two had unknown function, three were correlated to signal transduction mechanisms, two were correlated to translation, ribosomal structure and biogenesis, two were correlated to posttranslational modification, protein turnover, and chaperones, two were correlated to inorganic ion transport and metabolism, two were correlated to secondary metabolites biosynthesis, transport and catabolism and two were correlated to carbohydrate transport and metabolism. There was an additional gene correlated to lipid transport and metabolism, one correlated to the cytoskeleton, one correlated to coenzyme transport and metabolism, one correlated to energy production and conversion, one correlated to RNA processing and modification and one correlated to cell cycle control, cell division, and chromosome partitioning. In the GO analysis, 26 of the 43 had annotation information, among which, 21 were correlated to biological process, 21 were correlated to molecular function and 15 were correlated to cellular component.

## Discussion

### The characteristics of the method SLAF-seq

**Fig. 7:**
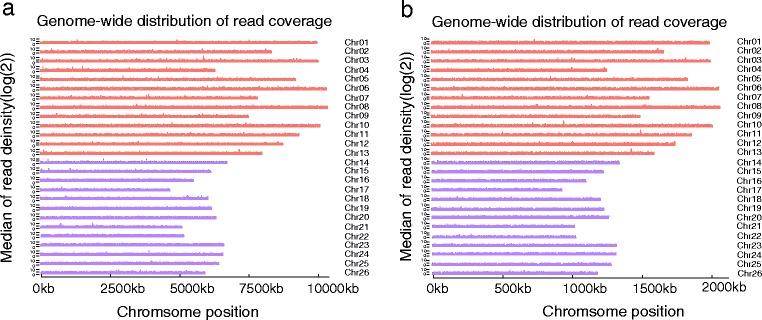
Genome-wide distribution of reads coverage. **a** Genome-wide distribution of reads coverage with window size of 10 K. **b** Genome-wide distribution of reads coverage with window size of 50 K

For the simplified genome sequencing, the key step was to make the simplified genome representative of the whole genome. This was completed through the election of suitable restriction endonuclease(s). When restriction endonuclease(s) were applied to the genome digestion and selected properly, the fragments generated by next-step sequencing would be a better representation of the genome. In the previous studies, usually a few common restriction endonucleases such as *Eco*RI, *Sbf*I and *Pst*I were used to digest the genome of various species [29]. Typically, only one restriction endonuclease was applied to the genome digestion [30–32]. The genome specificity of the species was ignored [29–33]. This might lead to uneven distribution of the selected fragments in the whole genome and thus make the simplified genome less representative. Eventually the number of markers developed and reliability of the genetic map might both be negatively affected [29, 33]. The SLAF-seq strategy, an effective NGS-based method for large-scale SNP discovery and genotyping, has been applied successfully in various species [12–14]. Compared with other tools for large-scale genotyping with NGS technology, such as RAD-seq and GBS, SLAF-seq displayed some unique superiorities. First, the pre-design scheme with different restriction endonuclease combinations was applied to simulate *in silico* the result script of endonuclease digestions based on the sequencing database of A, D and AD genomes of *Gossypium* [19, 34, 35] (Fig. 7). The information on genomic GC content, repeat conditions and genetic characteristics were referred to make up the digestion strategy. After two endonucleases combinations were applied to the genome digestion, the fragments ranging from 500 to 550 (including adapter) base pairs we harvested for sequencing create a better representation of the genome of *Gossypium hirsutum* L. Second, a dual-index will provide a higher sequence quality and more stable sequence depth among each sample, which is the key to developing high quality marker. Third, the marker underwent a series of dynamic processes to discard the suspicious markers during each cycle, until the average genotype quality score of all SLAF markers reached the cut-off value. As a result, the markers we developed might have a consistent distribution throughout the genome and the thus-built map might have a better coverage of the genome and be more reliable for the next step research activities.

### Genetic map construction

In previous studies, most of the genetic maps of cotton were based SSR markers. The low polymorphic rate of the SSR markers makes the SSR marker based maps unable to harbor a sufficient number of markers with a comparative poor coverage of the genome and low resolution. In most cases, these maps have large gaps, and sometimes the gap divides the chromosome into two or more linkage groups [16, 36, 37]. When the populations developed from interspecific crosses between G. *hirsutum* and G. *barbadense* were applied to the genetic map construction, the coverage and resolution of the map could be greatly improved [38–40]. However, the pragmatic applications of the genetic map developed from the interspecific populations have limited values as the polymorphic loci between G. *hirsutum* and G. *barbadense* may not show polymorphism within the cultivars of G. *hirsutum*. SNP markers could improve the coverage and resolution of the genetic map efficiently. Wang et al. [4] used SNP markers to construct a map through the RAD-seq, which harbored 3984 markers with a total distance of 3499.69 cM and an average distance of 0.88 cM. In our research, we constructed an HDGM through the SNP markers developed through the SLAF-seq method. Even though the map harbored a great number of markers and was more saturated than most of the previous ones, the total distance it covered was approximately the same as the previous studies. Some of the chromosomes only spanned very short genetic distances on the map. The shortest three chromosomes (chromosomes 15, 8 and 20) only spanned 41.39 cM, 45.12 cM and 48.44 cM, harboring 29, 56 and 60 markers respectively. Previous studies showed that different populations might generate varied chromosome genetic distances of the *Gossypium hirsutum* genome. In the initial steps of marker development through SLAF-seq, the quantities of SLAFs developed were about the same sizes in the different chromosomes. After several steps of screenings, the remaining numbers of SNPs for map construction varied greatly among the chromosomes, and the reduced number of remaining SNPs contributed to the shortness of some chromosomes. The collinearity comparison between the genetic map and the physical one validates the reliability of the constructed map. However, a better understanding of the genetic structure of these chromosomes might need an integrative analysis.

### The QTL of boll weight traits identification

Previous QTL studies were primarily focused on the fiber quality traits [1, 2, 40], while the research activities on yield traits especially the boll weight were seldom reported. The boll weight trait was significant and made a considerable contribution to the yield of cotton. Qin et al. [41] used the four-way cross (4WC) population to construct a map and identified only one QTL of boll weight on chromosome D2. The confidence interval of this QTL harbored three markers and spanned a distance of about 14.5 cM. Liu et al. [42] used RIL population to construct a map and identified the QTL of boll weight using the mean value of the data from four environments. Eighteen QTLs for boll weight were detected on 15 chromosomes. The confidence intervals of these QTLs harbored two or three markers. Yu et al. [43] used an interspecific backcross inbred line (BIL) population developed with a *G. hirsutum* and a *G. barbadense* to construct a genetic map and identified 10 QTLs on eight chromosomes (chromosomes 5, 11, 18, 21, 22, 24, 25, and 26). The confidence intervals of these QTLs also harbored two or three markers and spanned distances from 2 to 30 cM. In our study, we identified the QTL of the boll weight in 25 chromosomes except chromosome 8. Among them 16 QTLs were detected in at least three environments and were present on 11 chromosomes (chromosomes 1, 2, 3, 5, 7, 9, 13, 16, 22, 23, and 25 respectively). The confidence intervals of these QTLs harbored from two to 26 markers ranging from 0.7 to 13.9 cM. This implies that our results of QTL identification are more concise and accurate than previous studies and could be useful for future research looking at gene identification or cloning from these QTLs, or even breeding practices using MAS.

### The direction of the QTLs

Among the 16 stable QTLs that can be detected in at least three environments, eight had positive additive effects whereas the other eight had negative additive effects. This indicates that both the higher boll weight value parent sGK9708 and lower boll weight value parent 0–153 could contribute positive additive QTLs to increase the boll weight. This could be a possible factor behind the difference in the boll weight trait between the parents 0–153 and sGK9708. Theoretically, the greater the difference of one trait between the two parents, the higher the possibility that the positive additive effect of the QTLs would come from one parent. The RIL population was constructed primarily based on differences in fiber quality traits especially fiber strength between the parents 0–153 and sGK9708, therefore, the difference of fiber strength is larger than that of any other traits between 0–153 and sGK9708. In Sun’s report [16], seven QTLs of fiber strength were identified using this population, among which only one QTL had negative additive effects whereas the remaining six QTLs had positive additive effects. In Zhang’s report [17], seven QTLs of fiber strength on chromosome 25 were identified using the same population, all of which had a positive additive effect. In identifying the QTL clusters, the clusters that harbor all desired QTL alleles would make the greater contribution to the breeding practice when MAS is applied.

### Candidate gene functioning analysis

Among all 340 candidate genes being annotated in at least one channel of KOG, KEGG, and GO, some might be related to the boll weight trait. In KOG analysis, there were 21 function baskets. The posttranslational modification function, protein turnover, chaperones and signal transduction mechanisms harbored the largest number of candidate genes. Among the 44 genes located closest to the markers of genetic position, three genes *Gh_A07G1188*, *Gh_A07G1197*and *Gh_D09G1606* had a relation to signal transduction mechanisms. Two genes, *Gh_A05G1210* and *Gh_D04G1531* were related the function posttranslational modification, protein turnover, and chaperones. Two genes, *Gh_A07G1187* and *Gh_A13G0858,* had the translation function, ribosomal structure, and biogenesis, though this function basket did not harbor a large number of candidate genes. As the posttranslational modification, protein turnover and ribosomal structure were relative to the protein synthesis, it is probable that the genes correlated to this function contribute to the boll weight trait.

In KEEG analysis, the first three pathways which harbored the largest number of genes were plant hormone signal transduction, and protein processing in endoplasmic reticulum and ribosome, harboring six genes, four genes and four genes respectively. Of these 14 genes, three were located at the nearest positions of the markers, genetic position of which had the highest LOD values in the QTL mapping analysis. The gene *Gh_A13G0858* has a relationship to the ribosome, whereas genes *Gh_A13G0392* and *Gh_D06G0187* have a relationship to the plant hormone signal transduction. As the ribosome has a relationship to protein synthesis and some plant hormones such as auxin and gibberellin, these genes could contribute to the plant growth and eventually to the boll weight trait, particularly the gene *Gh_A13G0858*.

Although these genes were located the nearest position of the markers, genetic position of which had the highest LOD values in the QTL mapping analysis, but there still lacks direct evidence to prove that the function of these genes was correlated to the boll weight trait.

## Conclusions

This research reported the first HDGM of Upland Cotton (*Gossypium hirsutum*) with a RIL population using SNP markers developed by SLAF-seq. The HDGM had a total number of 5521 markers and a total distance of 3259.37 cM with an average marker interval of 0.78 cM. There were no gaps greater than 10 cM.We also identified QTLs of boll weight trait across 11 environments and identified candidate genes. Totally, 146 QTLs of boll weight was identified and 16 of them were detected in at least three environments with a stable QTL. Three hundred forty-four candidate genes were identified in the confidence intervals of stable QTLs and 44 of them were located in the nearest positions of the markers. The result of this research would provide information for the next phase of research such as fine mapping, gene functional analysis, pyramiding breeding and marker-assisted selection (MAS) as well.

### Availability of supporting data

The data sets supporting the results of this article are included within the article and its additional files.

## Additional files

Additional file 1: Table S1. Distribution of the SNP markers’ type on the genetic map. (XLSX 9 kb) Additional file 2: Table S2. The markers and their genetic distance in the genetic map. (XLSX 149 kb) Additional file 3: Table S3. The X^2^_value and P_value of all the markers in the genetic map. (XLSX 233 kb) Additional file 4: Table S4. The physical position of all the markers in the genetic map. (XLSX 213 kb) Additional file 5: Table S5. The genetic position of all the recombination hotspots in the genetic map. (XLSX 34 kb) Additional file 6: Table S6. All the QTLs identified including the ones that can be detected only in one environment. (XLSX 29 kb) Additional file 7: Figure S1. The physical map of the SNP markers and the candidate genes in the confidence intervals of the stable QTLs in A sub-genome. Footnote: Red: The candidate genes. Blue: The SNP markers. ★: The SNP markers that located in the nearest genetic position of the highest LOD value in QTL analysis. ●: The candidate genes that located in the nearest genetic position of the highest LOD value in QTL analysis. (PNG 959 kb) Additional file 8: Figure S2. The physical map of the markers and the candidate genes in the confidence intervals of the stable QTLs in D sub-genome. Footnote: Red: The candidate genes. Blue: The SNP markers. ★: The SNP markers that located in the nearest genetic position of the highest LOD value in QTL analysis. ●: The candidate genes that located in the nearest genetic position of the highest LOD value in QTL analysis. (PNG 827 kb) Additional file 9: Table S7. The GO annotation result of the candidate genes of the stable QTLs of cotton boll weight. (XLSX 13 kb) Additional file 10: Table S8. The KEGG annotation result of all the candidate genes of the stable QTLs of cotton boll weight. (XLSX 15 kb) Additional file 11: Table S9. The number of the candidate genes and the genes ID in each pathway in the KEGG annotation (XLSX 13 kb) Additional file 12: Table S10. The KOG annotation result all the candidate genes of the stable QTLs of cotton boll weight. (XLSX 15 kb) Additional file 13: Table S11. The number of the candidate genes in each function categories of the KOG annotation. (XLSX 11 kb)
