# Supplementary figures and images for: Construction of a high-density genetic map by specific locus amplified fragment sequencing (SLAF-seq) and its application to Quantitative Trait Loci (QTL) analysis for boll weight in upland cotton (Gossypium hirsutum.)
Source: BMC Plant Biol. 2016 Apr 11;16:79. doi: 10.1186/s12870-016-0741-4 (PMC4827241; doi:10.1186/s12870-016-0741-4)

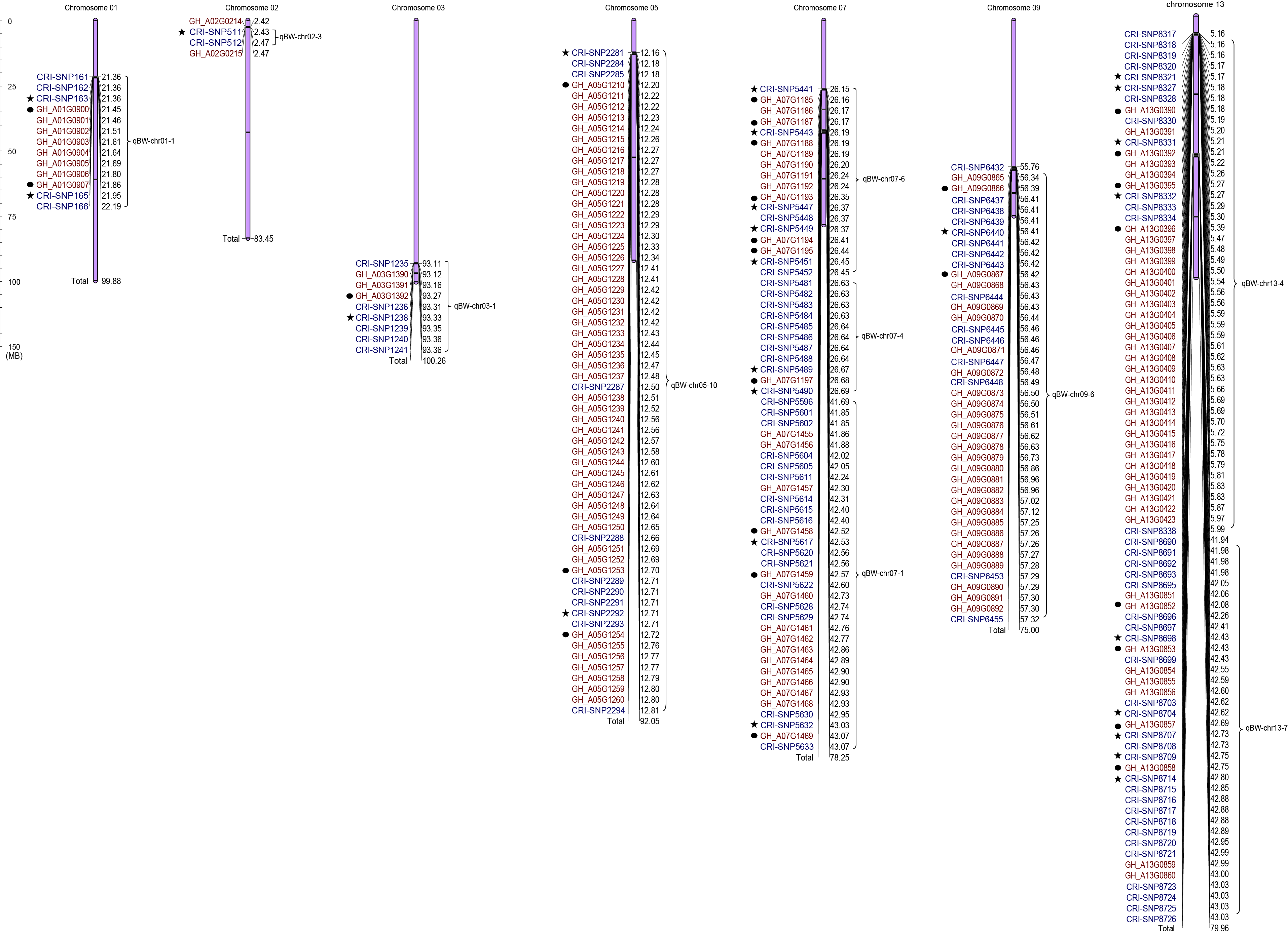

Supplement: Additional file 7: Figure S1. — The physical map of the SNP markers and the candidate genes in the confidence intervals of the stable QTLs in A sub-genome. Footnote: Red: The candidate genes. Blue: The SNP markers. ★: The SNP markers that located in the nearest genetic position of the highest LOD value in QTL analysis. ●: The candidate genes that located in the nearest genetic position of the highest LOD value in QTL analysis. (PNG 959 kb) [file 12870_2016_741_MOESM7_ESM.png]

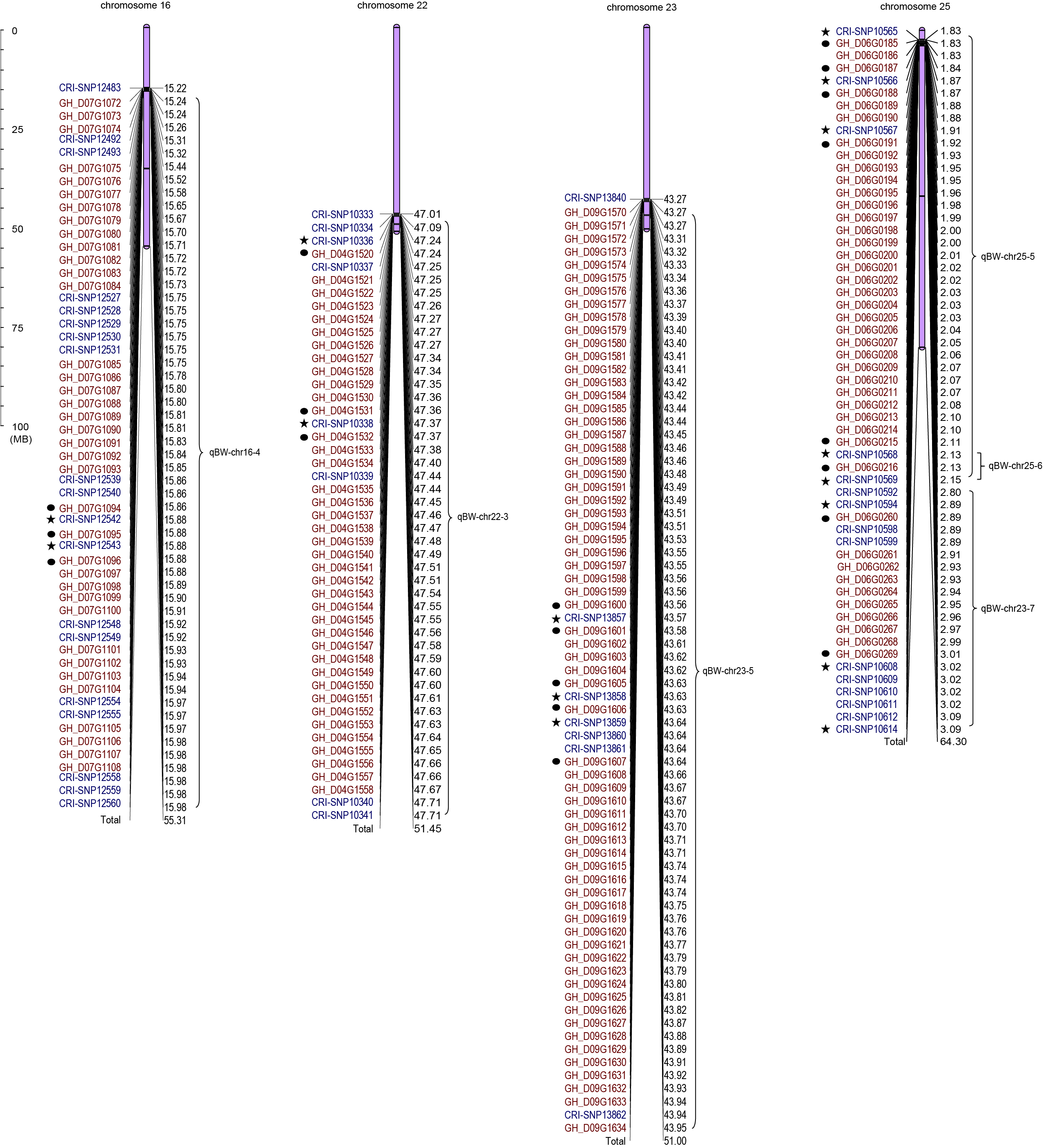

Supplement: Additional file 8: Figure S2. — The physical map of the markers and the candidate genes in the confidence intervals of the stable QTLs in D sub-genome. Footnote: Red: The candidate genes. Blue: The SNP markers. ★: The SNP markers that located in the nearest genetic position of the highest LOD value in QTL analysis. ●: The candidate genes that located in the nearest genetic position of the highest LOD value in QTL analysis. (PNG 827 kb) [file 12870_2016_741_MOESM8_ESM.png]
